# A quality-by-design approach to improve process understanding and optimise the production and quality of CAR-T cells in automated stirred-tank bioreactors

**DOI:** 10.3389/fimmu.2024.1335932

**Published:** 2024-04-09

**Authors:** Tiffany Hood, Fern Slingsby, Viktor Sandner, Winfried Geis, Timo Schmidberger, Nicola Bevan, Quentin Vicard, Julia Hengst, Pierre Springuel, Noushin Dianat, Qasim A. Rafiq

**Affiliations:** ^1^ Department of Biochemical Engineering, University College London, London, United Kingdom; ^2^ Product Excellence Bioreactor Technology, Sartorius Stedim UK Limited, Epsom, United Kingdom; ^3^ Digital Solutions, Sartorius Stedim Austria GmbH, Vienna, Austria; ^4^ Digital Solutions, Sartorius Stedim Biotech GmbH, Goettingen, Germany; ^5^ BioAnalytics Application Development, Essen BioScience Ltd. (Part of the Sartorius Group), Royston, United Kingdom; ^6^ Cell Culture Technology Marketing, Sartorius Stedim France S.A.S., Aubagne, France; ^7^ Cell Culture Technology Marketing, Sartorius Stedim Biotech GmbH, Goettingen, Germany

**Keywords:** immunotherapy, CAR-T, process understanding, quality-by-design, T cells, process optimisation, stirred-tank bioreactor

## Abstract

*Ex vivo* genetically-modified cellular immunotherapies, such as chimeric antigen receptor T cell (CAR-T) therapies, have generated significant clinical and commercial outcomes due to their unparalleled response rates against relapsed and refractory blood cancers. However, the development and scalable manufacture of these novel therapies remains challenging and further process understanding and optimisation is required to improve product quality and yield. In this study, we employ a quality-by-design (QbD) approach to systematically investigate the impact of critical process parameters (CPPs) during the expansion step on the critical quality attributes (CQAs) of CAR-T cells. Utilising the design of experiments (DOE) methodology, we investigated the impact of multiple CPPs, such as number of activations, culture seeding density, seed train time, and IL-2 concentration, on CAR-T CQAs including, cell yield, viability, metabolism, immunophenotype, T cell differentiation, exhaustion and CAR expression. Initial studies undertaken in G-Rex^®^ 24 multi-well plates demonstrated that the combination of a single activation step and a shorter, 3-day, seed train resulted in significant CAR-T yield and quality improvements, specifically a 3-fold increase in cell yield, a 30% reduction in exhaustion marker expression and more efficient metabolism when compared to a process involving 2 activation steps and a 7-day seed train. Similar findings were observed when the CPPs identified in the G-Rex^®^ multi-well plates studies were translated to a larger-scale automated, controlled stirred-tank bioreactor (Ambr^®^ 250 High Throughput) process. The single activation step and reduced seed train time resulted in a similar, significant improvement in CAR-T CQAs including cell yield, quality and metabolism in the Ambr^®^ 250 High Throughput bioreactor, thereby validating the findings of the small-scale studies and resulting in significant process understanding and improvements. This study provides a methodology for the systematic investigation of CAR-T CPPs and the findings demonstrate the scope and impact of enhanced process understanding for improved CAR-T production.

## 1 Introduction

The emergence of chimeric antigen receptor T cell therapies (CAR-T) have revolutionised the treatment for haematological malignancies. Despite their clinical success however, cost-effective, reproducible and robust manufacture of such therapies remains a significant translational and commercial bottleneck, and there is a need to address significant process variability (1a). With the price of commercially-available CAR-T therapies usually costing in excess of $300,000 per dose, it is critical that the underlying manufacturing and process issues associated with personalized therapies are addressed. This requires a deeper understanding of the impact of critical process parameter (CPPs) on the critical quality attributes (CQAs) of CAR-T cell therapies.

Significant differences in overall cell yield are often reported within the field and can be dependent upon the process conditions applied. For example, a study by 2 compared the effect of different medium formulations on overall T cell expansion, with expansion outcomes ranging from a 5- to 25-fold increase. Interestingly, some of the medium formulations demonstrated a wider range of fold expansion across donors while other medium formulations had more consistent expansion (2). Conversely, a study completed by 3 showed more consistent fold expansion when comparing different medium formulations but was able to achieve a much higher overall cell fold increase, >50, across all medium formulations (3). These studies illustrate the need to optimise the expansion protocol to understand what CPPs cause this wide range of expansion outcomes. This will ensure the process is less susceptible to changes in the variable starting material. Additionally, it is useful to understand what conditions improve overall robustness. Addressing both yield and robustness would help improve the manufacturing challenges of CAR-T cell therapies.

In addition to the variability associated with the incoming patient material, variability is also introduced by using uncontrolled, highly manual processes. For this reason, there has been a focus from regulatory agencies, such as the US Food and Drug Administration (FDA) and the Center for Biologics Evaluation and Research (CBER), on guidance emphasising the use of well-controlled manufacturing processes for the production of cell and gene therapy products to ensure consistent product quality and efficacy (4).

Implementing greater levels of automation and process control would address some of these challenges (5, 6). Many clinical trials have started trying to address this need for automation by utilising systems such as the CliniMACS Prodigy (6). The Prodigy system automates individual process steps which helps decrease the number of manual and open steps. However, significant product variability is still observed when using this system. 7 produced anywhere from about 1x10^9^ to 5 x10^9^ total cells after 9 days of culture in the Prodigy system (7). While the Prodigy automates process steps, there is still minimal sensor and process parameter control capabilities. Implementing more control into the process would likely improve overall process efficiency (8).

Stirred-tank bioreactors (STRs) are one type of expansion technology that could implement more control into the expansion process because process parameters such as pH, dissolved oxygen (DO), and agitation can be monitored and controlled. Additionally, STRs are well characterised and well understood manufacturing platforms as they are commonly used in the pharmaceutical industry (9). This allows for a faster transition into the platform.

The Ambr^®^ 250 High Throughput stirred-tank bioreactor platform is a high throughput, automated, multi-vessel 250mL system and has proven highly successful for process development for biopharmaceutical production (10–12), with a similar legacy for vaccine development (13), and more recently, cell and gene therapy applications (14–16). The Ambr^®^ 250 High Throughput presents a particularly attractive platform for CAR-T process development given its high throughput capability and scalability to GMP bioreactor platforms. Moreover, its automated liquid handling would be advantageous for the processing of multiple vessels simultaneously. We have previously investigated the use of the Ambr^®^ 250 High Throughput for suspension T cell and CAR-T production (15, 17) and have demonstrated its effectiveness and significant potential as a process development platform for CAR-T production in STRs. However, in these investigations, whilst they demonstrated proof-of-concept and the potential of using STRs for production, we noted the (15) scope for additional process understanding and optimisation to improve overall process efficiency.

In this present study, we focus on enhancing process understanding and optimisation to improve CAR-T cell yield and quality. Initial investigations used a design of experiments (DOE) approach to study a range of process parameters (**Table 1**) in a small-scale G-Rex^®^ multi-well plate format to understand their impact on CAR-T cell expansion and function, with a view to optimising the process. The DOE methodology provides a systematic approach to study the impact of each growth parameter and its concomitant effect on the other investigated parameters, thereby enabling a larger design testing space to be investigated so a more representative optimum can be identified when compared with 1-factor at a time studies. The second phase of the study focused on investigating the optimised and pre-optimised conditions on three additional healthy donors to determine whether the results obtained from the first phase of the DOE translated across donors. The final phase sought to validate these optimised conditions identified by the small-scale studies and apply this to a larger-scale STR bioprocess using the Ambr^®^ 250 High Throughput bioreactor.

**Table 1:** Factors and levels investigated in a small-scale DOE in G-Rex^®^ with CAR-T cells.

Factors		Levels		Factor Rationale
**Number of Activations**	1		2	Maintaining growth versus over-activation
**Seed Train Time** (days)	3	5	7	Initial growth lag versus primary cell exponential growth limits
**Seeding Density** (cells/cm2)	0.25x10^6^	0.5x10^6^	0.75x10^6^	Positive cell signalling from cells being too sparse vs negative cell signalling from cells being too dense
**IL-2** (IU/mL)	30	65	100	Activation versus potential over activation

## 2 Materials and methods

### 2.1 CAR lentivirus

The CAR lentivirus was produced as previously described (17). After the lentivirus was produced, the functional titre was assessed using an infectivity assay (18).

### 2.2 Human primary T cells

Primary T cells used in these studies were isolated from healthy, human donors from whole blood (Cambridge Bioscience, UK) or leukopak (BioIVT, West Sussex, UK) samples. The cells were delivered the same day as the collection and processed immediately upon delivery to isolate the CD3+ T cells.

For cells isolated from whole blood, the peripheral blood mononuclear cells (PBMCs) were isolated as described previously (15). For cells isolated from leukopaks (BioIVT, West Sussex, UK), the cells were washed 1:1 in MACS^®^ wash buffer (Miltenyi Biotec, Surrey, UK) prepared according to the manufacturer’s specifications. CD3+ T cells were isolated using the human Pan T isolation kit (Miltenyi Biotec Ltd., UK) according to the manufacturer protocol. After processing, the cells were immediately cryopreserved at 50e^6^ cells/mL in CryoStor^®^ (STEMCELL Technologies UK Ltd, UK).

### 2.3 Primary T cell culture

The primary cell culture medium used in all experiments consisted of Roswell Park Memorial Institute (RPMI) 1640 (Gibco™, Thermo Fisher Scientific, UK), 10% fetal bovine serum (FBS, Gibco™, Thermo Fisher Scientific, UK), 2mM L-glutamine (Gibco™, Thermo Fisher Scientific, UK), and 1% Antibiotic-Antimycotic (100X, Gibco™, Thermo Fisher Scientific, UK). Primary T cell medium was also supplemented with Interleukin-2 (IL-2, research grade, Miltenyi Biotec, Surrey, UK) as specified.

Cells were thawed at 2x10^6^ cells/mL in T cell complete medium in suspension cell culture flasks (Sarstedt AG & Co. KG, Germany). One day after thaw, the T cells were activated at 1:1 ratio of cells to Magnetic Dynabeads^®^ (Thermo Fisher Scientific Inc., Waltham, MA, USA) according to the manufacturer protocol with a final cell density of 1x10^6^ cells/mL. Research-grade IL-2 (Miltenyi Biotec Ltd., UK) was added to the flasks at the desired experimental concentration of 30, 65 or 100 IU/mL at the time of activation.

One day after activation, the T cells were transduced with the CAR lentivirus using a multiplicity of infection (MOI) of 3 in 6-well suspension cell culture plates (Sarstedt AG & Co. KG, Germany) coated with RetroNectin^®^ Recombinant Human Fibronectin Fragment (Takara Bio Inc., France). The plates were spinnoculated by centrifugation at 1,000g for 40 minutes. One day after transduction, cells were washed and plated at 0.5x10^6^ cells/mL for the seed train cell expansion lasting 3, 5 or 7 days.

### 2.4 Cell expansion

After cell activation, transduction, and the seed train cell expansion, experiments were initiated and referred to as day 0 of the expansion phase. At the start of the expansion phase, cells were plated in fresh medium and IL-2 at the experimental concentration, and for conditions requiring a second activation, Dynabeads^®^ were added at a 1:1 cell to bead ratio. Unless otherwise stated, daily 1mL samples were taken for cell counts and metabolite analysis. After 7 days of expansion samples were analysed by flow cytometry.

For expansion in G-Rex^®^ 24 well plates (Wilson Wolf Manufacturing, New Brighton, MN) cells were seeded at the experimental seeding density of 0.25, 0.5 or 0.75 x10^6^ cells/mL. On days 3 and 5 of expansion, fresh IL-2 was added at the desired experimental concentration based on the volume of each well. Cells were counted on days 0 and 7 of the expansion.

For flask expansion, cells were seeded at 0.5x10^6^ cells/mL and fed every other day by diluting the culture down to 0.5x10^6^ cells/mL with T cell growth medium. Fresh IL-2 was added based of the cell culture volume post feed.

Stirred tank bioreactor expansion processes were completed using a two-bioreactor Ambr^®^ 250 High Throughput system (Sartorius, UK) and unbaffled, single-impeller Ambr^®^ 250 vessels (16). The bioreactor process was based on that described by 17. The bioreactor parameters were controlled at 37°C, 200rpm, 7.15 pH, and 50% dissolved oxygen (DO).

### 2.5 T Cell analytics

#### 2.5.1 Cell counting and viability

Cell density and viability were measured using the NucleoCounter^®^ NC-3000™ (ChemoMetec A/S^©^, Denmark) with Via-1-Cassettes™ (ChemoMetec A/S^©^, Denmark) according to the manufacturer’s protocol.

#### 2.5.2 Metabolite analysis

Medium samples were analysed using the CuBiAn HT270 bioanalyzer (Optocell GmbH & Co, KG, Germany) to determine levels of glucose, glutamine, ammonia, and lactate concentrations. Prior to measurement, the samples were centrifuged at 350g for 5 minutes and the supernatant was frozen at -80°C.

#### 2.5.3 Flow cytometry

Phenotypic cell characteristics were analysed via flow cytometry on fresh cell samples on the day of sampling using the BD LSRFortessa X-20 flow cytometer (BD Biosciences, UK). Cells were stained for CD3-BUV395, CD4-BUV805, CD8- APC-Cy7, CCR7-BV421, CD45RO-PE-Cy7, CD56-BV605, CD34(CAR)-AlexaFluor647, CD69-FITC, PD-1-PE, LAG-3- BV711, and Live/Dead-UV511. A minimum of 50,000 events were recorded for all conditions. Gates were confirmed based on FMO (fluorescence minus one) controls for CCR7, CD45RO, CD56, CD69, PD-1, and LAG-3.

#### 2.5.4 *In vitro* killing assay

The *in vitro* killing assay was evaluated using the Incucyte^®^ S3 live-cell analysis platform (Sartorius, UK) according to the manufacturer protocol. A 1:1 target to effector ratio co-culture was used with GFP-positive, CD19-positive NALM6 target cells. One day after the experimental effector cells were thawed, the CAR positive T cells were isolated using a human CD34 MicroBead isolation kit (Miltenyi Biotec Ltd., UK). During the co-culture, the target cell count was normalized to initial cell plating via the Incucyte^®^ analysis software.

Cytokine analysis for tumour necrosis factor-alpha (TNF-α) and interferon-gamma (IFN-γ) was completed on the spent medium from the final timepoint of the killing assay using the iQue^®^ Qbeads from the iQue^®^T cell Activation Cell and Cytokine Profiling Kit (Sartorius, UK). The samples were analysed using the iQue^®^ Advanced flow cytometer (Sartorius, UK).

### 2.6 Equations

#### 2.6.1 Cell growth calculations

The population doublings of cells were calculated based on the equation used by 19 (19).


$$PD_{n}=\frac{1}{\ln2}*\;\ln\frac{C_{x,\;n}}{C_{x,\;n-1}}$$

Equation 0.1 Population doublings were calculated where PD_n_ was population doublings between time t_n_ and t_n-1_, and C_x, n_ and C_x, n-1_ were the cell number at times t_n_ and t_n-1_, respectively.


$$cPD_{n}=PD_{n-1}+PD_{n}$$

Equation 0.2 Cumulative population doublings was calculated where cPD_n_ was cumulative population doublings at time t_n_, and PD_n-1_ and PD_n_ were the population doublings at times t_n_ and t_n-1_, respectively.

The cell fold increase during the expansion phase was calculated based on the population doublings achieved.


$$Cell\;Fold\;Increase=2^{cPD_{n}}$$

Equation 0.3 Cell fold was calculation where cPD_n_ was the cumulative population doublings at time t_n_.

The specific growth was calculated as previously described (15).

#### 2.6.2 Metabolite calculations

Average metabolite concentrations were calculated over the exponential phase of the expansion phase from days 2-7 (15).

**Glucose and glutamine specific consumption**

The specific glucose and glutamine consumption rates were calculated based on equations used by 20 and 15 (20).


$$q_{met,n}=\left(\frac{\mu_{n}}{C_{x,\;n-1}}\right)*\left(\frac{\left(c_{met,\;n-1}*\;\left(1-D_{n}\right)+\left(m_{x}*D_{n}\right)\right)-c_{met,\;n}}{e^{\mu \left(t_{n}-t_{n-1}\right)}-1}\right)$$

Equation 0.4 Metabolite specific consumption was calculated where met was the metabolite, glucose or glutamine of interest, q_met, n_ was the specific consumption rate, μ is the specific growth rate (day^-1^) between time t_n_ (day) and t_n-1_ (day), C_x, n-1_ was the cell number at time t_n_, c_met, n_ and c_met, n-1_ are the concentration of the metabolite of interest at time t_n_ and t_n-1_, respectively, m_x_ was the concentration of the metabolite in fresh feed medium, and D_n_ is the daily perfusion rate if perfusion is active.

The specific lactate production and ammonia production were calculated based on equations used by 20 and 15.


$$p_{met,n}=\left(\frac{\mu }{C_{x,\;n-1}}\right)*\left(\frac{c_{met,\;n}-\left(c_{met,\;n-1}*\;\left(1-D_{n}\right)\right)}{e^{\mu \left(t_{n}-t_{n-1}\right)}-1}\right)$$

Equation 0.5 Metabolite specific production was calculated where ‘met’ was the metabolite, lactate or ammonia, of interest, p_met,n_ was the specific production rate, μ was the specific growth rate (day^-1^) between time t_n_ (day) and t_n-1_ (day), C_x, n-1_ was the cell number at time t_n_, c_met, n_ and c_met, n-1_ were the concentration of the metabolite of interest at time t_n_ and t_n-1_, respectively, m_x_ was the concentration of the metabolite in fresh feed medium, and D_n_ was the daily perfusion rate if perfusion was active.

The yield of lactate from glucose was calculated to determine the ratio of lactate molecules produced to glucose molecules consumed.


$$\frac{dLac}{dGluc}=\;\frac{p_{lac,\;n}-p_{lac,\;n-1}}{q_{gluc,\;n}-q_{gluc,\;n-1}}$$

Equation 0.6 dLac/dGluc was calculated where $\frac{\text{dLac}}{\text{dGluc}}$ was the yield of lactate from glucose, P_lac, n_ and P_lac, n-1_ was the specific lactate production rate at times t_n_, which represents the final timepoint (day) and t_n-1_ which represents the initial timepoint (day), respectively.

### 2.7 Design of experiments regression modelling

The statistical models for the experimental responses were analysed using MODDE^®^ v13 (Sartorius, Göttingen, Germany). The model analysis included an analysis of the importance of model coefficients. All coefficients included within the models had a p-value<0.05 (unless they were included to hierarchy for interaction terms), indicating their inclusion in the model was significant. The overall model accuracy and fit for purpose was assessed by looking at the descriptive power (indicated by a high R²) and the predictive power (indicated by a high Q²). In addition, the difference between the two should not be greater than 0.2 which would indicate a too optimistic model.

### 2.8 Statistical analysis

Statistical analysis was completed using Jmp^®^ software (SAS Institute Inc., NC, USA). Statistical tests were based on criteria of statistical methods outlined by Shingala et al., 2015 (21). Prior to any statistical comparisons the distribution of the data was analysed for normality and the variance of the data was compared. The variance of the means was determined using the appropriate statistical method of Brown-Forsythe, Levene, or Bartlett. A comparison of the means was completed by an ANOVA or Welch’s ANOVA and, if applicable, a post-hoc pairwise comparison using Tuckey-Kramer, Steel Dwass, or Dunn pairwise, was then completed. Values for these tests were considered statistically significant when the p-values were less than 0.05 (*), 0.01 (**), 0.001 (***), or 0.0001 (****).

### 2.9 Figures and graphics

Summary diagrams in this report were created with BioRender.com (BioRender, Toronto, Ontario). Line charts and bar graphs were completed using GraphPad Prism (GraphPad, La Jolla, USA). For the DOE design generation and analysis d MODDE 13.0.2. (Sartorius, Göttingen, Germany) was used.

## 3 Results

### 3.1 Design of experiments study to investigate impact of process parameters on CAR-T cell yield, quality and metabolism

To determine the effect and interactions of potential critical process parameters on final CAR-T cell yield and quality, a DOE study was performed investigating the following process parameters: (1) number of activations, (2) culture seeding density, (3) seed train time and (4) interleukin-2 (IL-2) concentration. A summary of these process parameters, including the range of levels for each factor and the rationale for selection is summarised in **Table 1**. Most processes currently activate the cells at the start of culture to initiate T cell growth (3, 22). However, some protocols re-activate the cells during culture to maintain growth (15, 23). By activating the cells a second time, this could enable the cells to continue growing longer (15). However, chronic activation has also been shown to cause T cells to become exhausted (24). Therefore, the balance between these aspects should be understood so this experiment tested the effect of one activation versus the inclusion of a second activation.

Current clinical protocols have a broad range of expansion times, sometimes even going beyond 30 days (1b;^25^). Seed train lengths of 3, 5, and 7 days were selected to understand the impact of expanding cells longer. Growing the cells longer could enable a higher yield, however T cells are primary cells and therefore are limited in growth so the impact of these aspects must be understood (1b;^25^).

Seeding densities above and below the manufacturer recommended 0.5x10^6^ cells/cm^2^ were chosen for testing (G-Rex^®^ Instructions for Use). A higher seeding density could enable higher yield at the end of culture. A lower seeding density, on the other hand, could enable faster growth as there would be more available nutrients and potentially fewer secreted cell signal from neighbouring cells that could inhibit cell growth (26).

Interleukin 2 (IL-2) is a growth factor that is supplemented into T cell cultures to activate T cells and encourage growth (27). However, chronic stimulation of growth of T cells can cause T cells to become exhausted and dysfunctional (24). Therefore, a range of IL-2 concentrations were selected with a particular focus on concentrations between 30 IU/mL and 100 IU/mL. Most T cell growth protocols utilising IL-2 seem to fall in this range (22, 28–30).

The DOE design for this optimisation experiment ensured the test conditions selected had good coverage within the DOE factor design space (**Supplementary Figure 1**).

**Figure 1:**
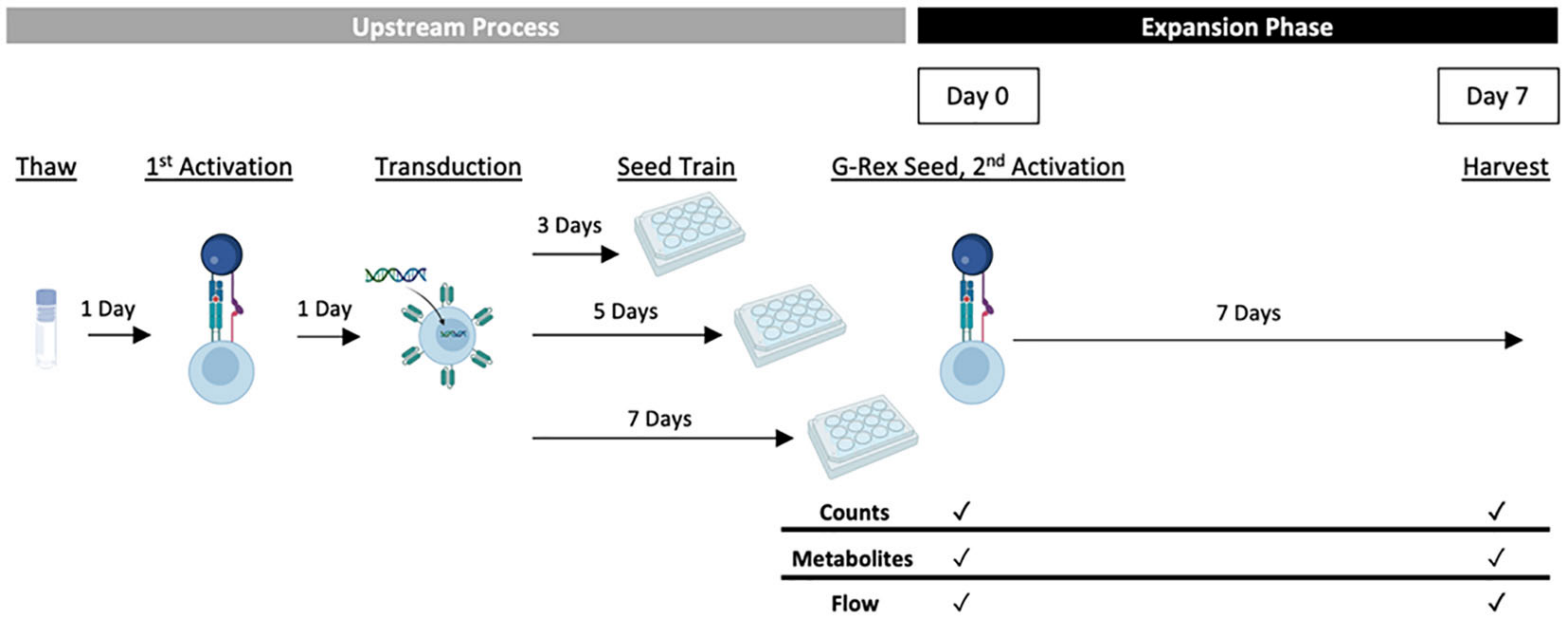
Experimental CAR-T expansion G-Rex^®^ DOE overview. Cells thawed from one donor were activated a day later using Dynabeads^®^ and the experimental IL-2 concentration (30, 65 or 100 IU/mL). The following day, all conditions were transduced with anti-CD19 CAR lentivirus and pre-expanded for the experimental seed train lengths (3, 5 or 7 days). Pre-expanded cells were then seeded into G-Rex^®^ 24-well plates at varied seeding densities (0.25, 0.5 or 0.75 x 10^6^ cells/mL) and expanded for 7 days with monitored cell growth and quality. For conditions with a second activation step, Dynabeads^®^ were added before G-Rex^®^ seeding.

The responses measured as part of the DOE included a range of CAR-T cell growth and quality attributes that are summarised in **Table 2**. In order to analyse these characteristics, statistical models describing the effect of the process parameters on each response were created. One donor (HD1) was used to test all conditions in G-Rex^®^ 24-well well plates to understand the effects of just the experimental factors. An overview of the experimental design is presented in **Figure 1**.

**Table 2:** Responses measured in a small-scale DOE in G-Rex^®^ with CAR-T.

Response	Measurement	Response Rationale
Cell Growth	Cells/mL	• Represents cell yield-essential to achieve CAR-T dose
Viability	Percentage viable cells	• Cell yield often based on viable cells • Minimum viability often CAR-T product release criteria
Metabolites	Glucose, lactate, glutamine, ammonia	• Shows nutrient availability in medium • Consumption and production indicative of cell state and metabolism
Phenotype	CD3, CD4, CD8	• Cell type
Memory Differentiation	CCR7, CD45RO	• Less differentiated cells associated with CAR-T clinical efficacy
Activation	CD69	• Indicative of cell state
Exhaustion	LAG3, PD1	• Less exhausted cells associated with CAR-T clinical efficacy
CAR Expression	CAR marker	• CAR-T dosage often based on CAR+ cells

#### 3.1.1 Impact of process parameters on CAR-T cell yield

**Figure 2:**
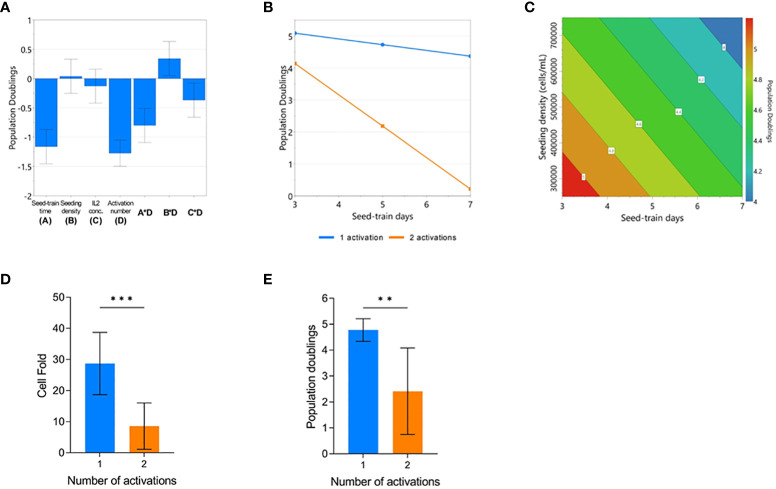
Repeated activation, higher seeding density, and longer seed train time negatively impact CAR-T cell yield. Regression coefficients included in the modelled response for population doublings (PD) on day 7 of G-Rex culture **(A)**. Modelled factor-interaction between seed-train time and number of activations **(B)**. Modelled effects of seeding density and seed-train time on PD, assuming 1 activation and 30 IU/mL of IL2 **(C)**. Effect of number of activations on cell fold **(D)**, and PD **(E)**. Error bars represent standard deviation. Statistical pooled t-test analyses completed where ** is p<0.01, *** is p<0.001.

The impact of process parameters on cumulative population doublings (cPD) were modelled using the DOE design and the resulting model (p<0.001, R2 = 0.966, Q2 = 0.868). The number of activations, seed train time, seeding density and IL-2 concentration were all found to significantly impact cPD (**Supplementary Figure 2**). Plotting the effects of each model co-efficient revealed that the number of activations resulted in the largest, adverse impact on cell yield (**Figure 2A**). An interaction effect between the number of activations and seeding density was also found to significantly contribute towards final cPD. Specifically, higher seed train times led to decreased cPD, most particularly for conditions that were activated twice (**Figure 2B**). The possibility of identifying such interactions between process parameters represents one of the key advantages of using a DOE approach.

When looking further into the factors that impacted cPD when the cells were only activated once, both a higher seed train time and higher culture seeding density had a detrimental effect on cell yield resulting in decreased growth (**Figure 2C**). On average, conditions that were activated once achieved a cell fold of 28.7 ± 10 whereas conditions that were activated twice resulted in a cell fold of 8.6 ± 7.4 after 7 days of culture in the G-Rex^®^ plate (p=0.0008) (**Figure 2D**). This trend was also observed for cPD with conditions activated once reaching 4.8 ± 0.4 cPD versus 2.4 ± 1.6 cPD for conditions that were activated twice (p=0.0008) (**Figure 2E**).

#### 3.1.2 Impact of process parameters on CAR-T cell quality

**Figure 3:**
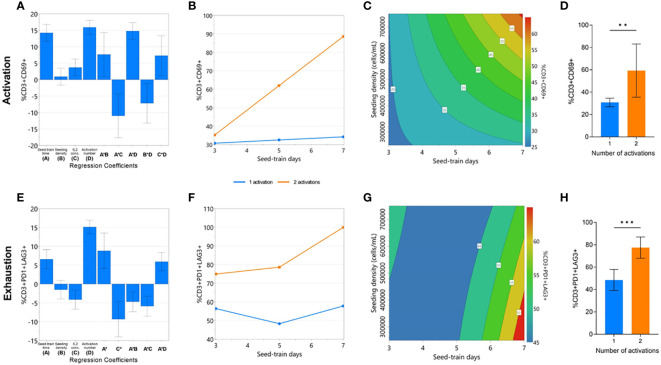
Cell quality characteristics impacted by repeated activation and longer seed train time. The effects of the experimental factors on CD3+CD69+ and CD3+PD1+LAG3+ expression on day 7 of G-Rex^®^ culture were modelled: significant regression coefficients plot **(A, E)**, factor-factor interaction plot **(B, F)**, and contour plot of modelled response **(C, G)**. The effect of number of activations on mean CD3+CD69+ and CD3+PD1+LAG3+ expression **(D, H)**. Statistical pooled t-test analyses completed where ** is p<0.01, *** is p<0.001.

Quality attributes associated with cell function, such as cell activation, exhaustion, and differentiation, must also be considered at the end of CAR-T cell expansion. Although the factors investigated in this DOE were not found to impact cell differentiation, significant models could be generated for cell activation (p<0.0001, R2 = 0.984, Q2 = 0.749) and exhaustion responses (p<0.0001, R2 = 0.933, Q2 = 0.317) (**Supplementary Figure 3**, **4**). All investigated process parameters were included in each model, and the number of activations was again found to have the greatest effect, on both activation and exhaustion responses (**Figure 3A, E**). Significant interaction effects between seed train time and the number of activations were again found in both modelled responses.

A second activation step resulted in a drastically higher percentage of CD69+ cells after longer seed train times, but not following shorted seed train times (**Figure 3B**). In conditions with a single activation step, longer seed train time and higher cell densities were both associated with a higher percentage of CD69+ cells (**Figure 3C**). On average, conditions with a single activation yielded significantly less CD69+ cells (30.84 ± 3.8%) than those with a double activation (59.36 ± 23.8%) (p<0.01) (**Figure 3D**).

Similarly, expression of both exhaustion markers was also found to significantly increase after longer seed train times. This effect was further pronounced when the cells were activated twice (**Figure 3F**). In conditions with a single activation step, longer seed train times alongside lower seeding densities were associated with higher expression of exhaustion markers (**Figure 3G**). Overall, conditions only activated once had lower dual expression of exhaustion makers PD-1 and LAG3 at the end of culture with 48.5 ± 9.5% of cells expressing PD1 and LAG3, compared to 77.5 ± 9.5% PD1+LAG3+ expression for conditions activated twice (p=0.0006) (**Figure 3H**).

#### 3.1.3 Impact of process parameters on CAR-T cell metabolism

**Figure 4:**
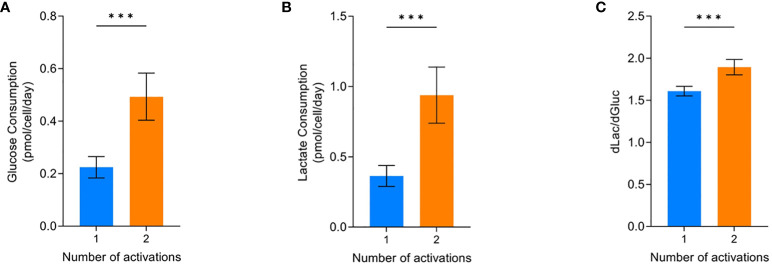
Effect of activations on the average glucose consumption **(A)**, lactate production **(B)**, and dlac/dgluc **(C)**. Error bars represent standard deviation. Statistical pooled t-test analyses completed where *** is p<0.001. Dashed lines represent the confidence interval of the model. All model graphs shown represent the model when IL-2 = 30 IU/mL and seeding density = 2.5x10^6^ cells/cm^2^.

The glucose consumption and lactate production of the cells were most affected by the two activations condition. Conditions that were activated twice and grew less (**Figure 2B**) had significantly higher glucose consumption (p=0.0004) and lactate production (p=0.0004) compared to cells that were only activated once (**Figure 4A, B**). Analysing the amount of lactate produced from glucose can be indicative of the metabolic processes the cells are utilising. Cells activated twice produced 1.89 ± 0.09 lactate molecules for every glucose molecule consumed. This was significantly higher (p=0.0004) than the 1.61 ± 0.06 lactate molecules produced per glucose molecule by cells that were only activated once (**Figure 4C**).

If cells are utilising glycolysis, 2mol ATP and 2mol of lactate will be produced per 1mol glucose. Whereas, oxidative phosphorylation will completely break down glucose into CO_2_ and produce 36mol ATP per mol of glucose (31, 32). Therefore, it is likely the conditions only activated once had increased levels of the more energy efficient oxidative phosphorylation pathway since the amount of lactate produced was below the theoretical level of 2 for pure glycolysis.

#### 3.1.4 Optimised CAR-T cell process parameters

After analysing the individual responses measured in the DOE, it is important to look at the effects of the parameters more holistically (**Table 3**). With an optimised end product defined to be one that exhibits high cell yield with low CD3+PD1+LAG3+ exhaustion marker expression, it was found that a process which is comprised of a single activation step and a 3-day seed train was the optimal levels from the process parameters investigated, based on the DOE models for each experimental response. The concentration of IL-2 was not shown to have a significant impact on the optimum. As the IL-2 concentration did not appear to have any significant impact, it was decided to use a lower concentration of IL-2 with a view to minimising culture costs should the process be performed at a larger scale (**Table 3**). In summary, the optimised process that was validated for subsequent studies was comprised of a single activation step, 3-dayseed train and supplementation of 30IU/mL IL-2.

**Table 3:** Summary of the effects of the experimental factors on key responses.

Response	Desired Effect	↓ Activations	↓ Seed Train (days)	↓ Seeding Density (cells/cm^2^)	↓ IL-2 (IU/mL)
Growth	maximize	**+++**	**+**	**+**	
Exhaustion marker expression	minimize	**+++**	**+**		
Glucose consumption per cell	minimize	**+++**	**+**		
Lactate production per cell	minimize	**+++**	**+**		
**Optimised Parameters**		1	3	0.25	30-100
**Sub-optimised Parameters**		2	7	0.75	30-100

+++ represents a strong effect, +represents a moderate effect, blank represents no effect. A desirable outcome was classified as maximising cell fold and minimising CD3+PD1+LAG3+ exhaustion marker expression to determine the optimised and sub-optimised process parameter levels.

### 3.2 Assessment of optimised process on CAR-T yield and quality with multiple donors

The previous study was conducted with a single healthy donor. However, a significant challenge for CAR-T production is the innate donor-to-donor variability. To therefore understand the robustness of the identified process effects, the optimised conditions established from the previous study were tested on three different healthy donors (HD 1-3) in flask cultures. The optimised and pre-optimised process were set using parameter settings identified in the previous G-Rex^®^ DOE that correlated with the highest and lowest cell yield and quality, respectively (**Table 3**). As the findings from the previous study demonstrated that the number of activations and seed train time had the greatest impact on cell yield, quality and metabolism, the IL-2 concentration and the seeding density were held constant at 30IU/mL and 0.5cells/mL, respectively for both the optimised and pre-optimised process.

**Figure 5:**
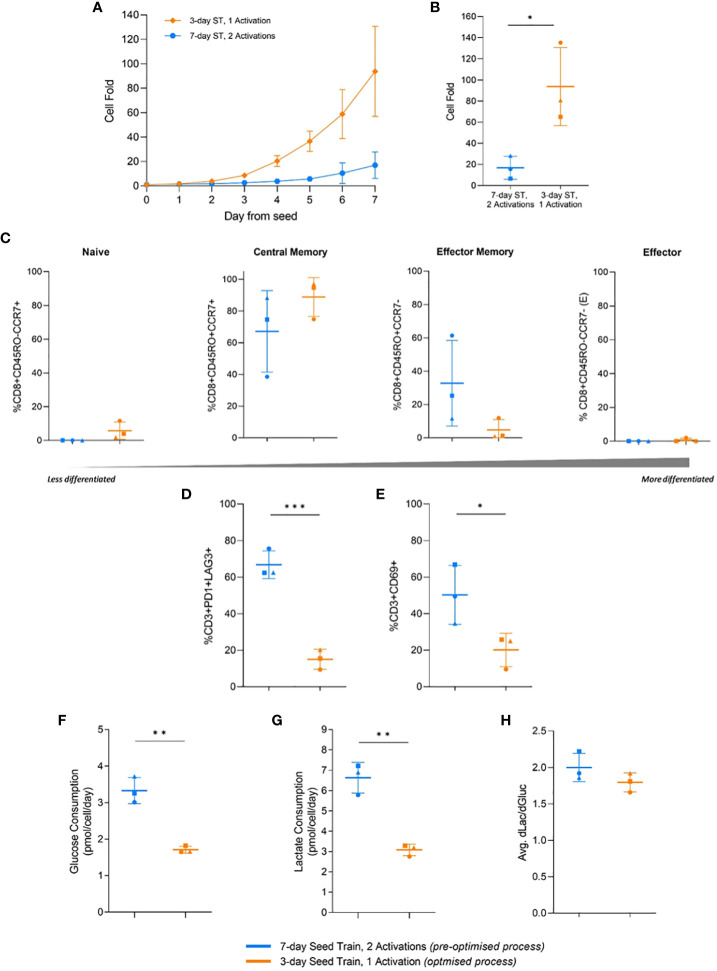
Effects of process parameters translated to flasks and multiple donors. Cell fold in flask culture by day **(A)**, and on day 7 **(B)**. T-cell differentiation **(C)**, exhaustion **(D)**, and activation **(E)** on day 7 of flask culture. Average glucose consumption **(F)**, lactate production **(G)**, and change in lactate divided by change in glucose (dLac/dGluc) **(H)**. Day 0 represents the day flasks were seeded post seed train (ST). Points represent each donor (square = HD1, triangle = HD2, circle = HD3). Error bars represent standard deviation. Statistical pooled t-test analyses completed where * is p<0.05, ** is p<0.01, *** is p<0.001.

In the flask cultures, the optimised process with a 3-day seed train and single activation step achieved significantly higher (p=0.026) cell fold at the end of culture, in alignment with the previous DOE findings (**Figure 5B**). This increased cell growth was observed as early as day 2 after the flasks were seeded (**Figure 5A**). By the end of the culture, the pre-optimised process with a 7-day seed train and two activations achieved a cell fold of 16.9 ± 10.9 while the optimised process achieved a much higher cell fold of 93.8 ± 36.9 (**Figure 5B**).

No significant difference (p>0.05) in the memory differentiation populations was observed between the optimised and pre-optimised processes. The majority of cells were in a less differentiated central memory state at the end of culture, which is associated with improve CAR-T clinical efficacy (**Figure 5C**) (33).

In this study, the pre-optimised process resulted in 66.8 ± 7.6% of T cells expressing both PD1 and LAG3, while the optimised process had significantly less expression (p=0.0007), with only 15.1 ± 5.5% of T cells expressing these two exhaustion markers (**Figure 5D**). The pre-optimised condition in this experiment showed 50.3 ± 16.1% CD3+CD69+ expression, while the optimised condition had lower (p=0.048) CD3+CD69+ expression, 20.1 ± 9.2% (**Figure 5E**). The simultaneous higher expression of PD1, LAG3, and CD69 in the pre-optimised condition aligned with the findings of the previous DOE.

Moreover, the pre-optimised condition also consumed significantly higher (p=0.0016) amounts of glucose per cell, nearly double, and produced more (p=0.016) lactate per cell compared to the more optimised condition (**Figure 5F, G**). However, there was no significant difference in the ratio of lactate molecules produced per glucose molecule consumed between the two conditions. The pre-optimised condition had a ratio of 2.0 ± 0.2 and the optimised condition had a ratio of 1.8 ± 0.1 (**Figure 5H**). Therefore, both conditions are likely primarily using glycolysis for the breakdown of glucose.

### 3.3 Assessment of optimised process on *in vitro* CAR-T cytotoxicity with multiple donors

Having demonstrated the robustness of the findings across multiple healthy donors, the impact of the optimised process on the CAR-T cell *in vitro* cytotoxic capability was investigated. An *in vitro* killing assay was performed using the CAR-T cells generated using the optimised process in culture with the target NALM6 cells. The number of NALM6 target cells were tracked over time and normalized to the initial seeding density, where anything above the normalized value of 1 indicated growth of the target cells.

**Figure 6:**
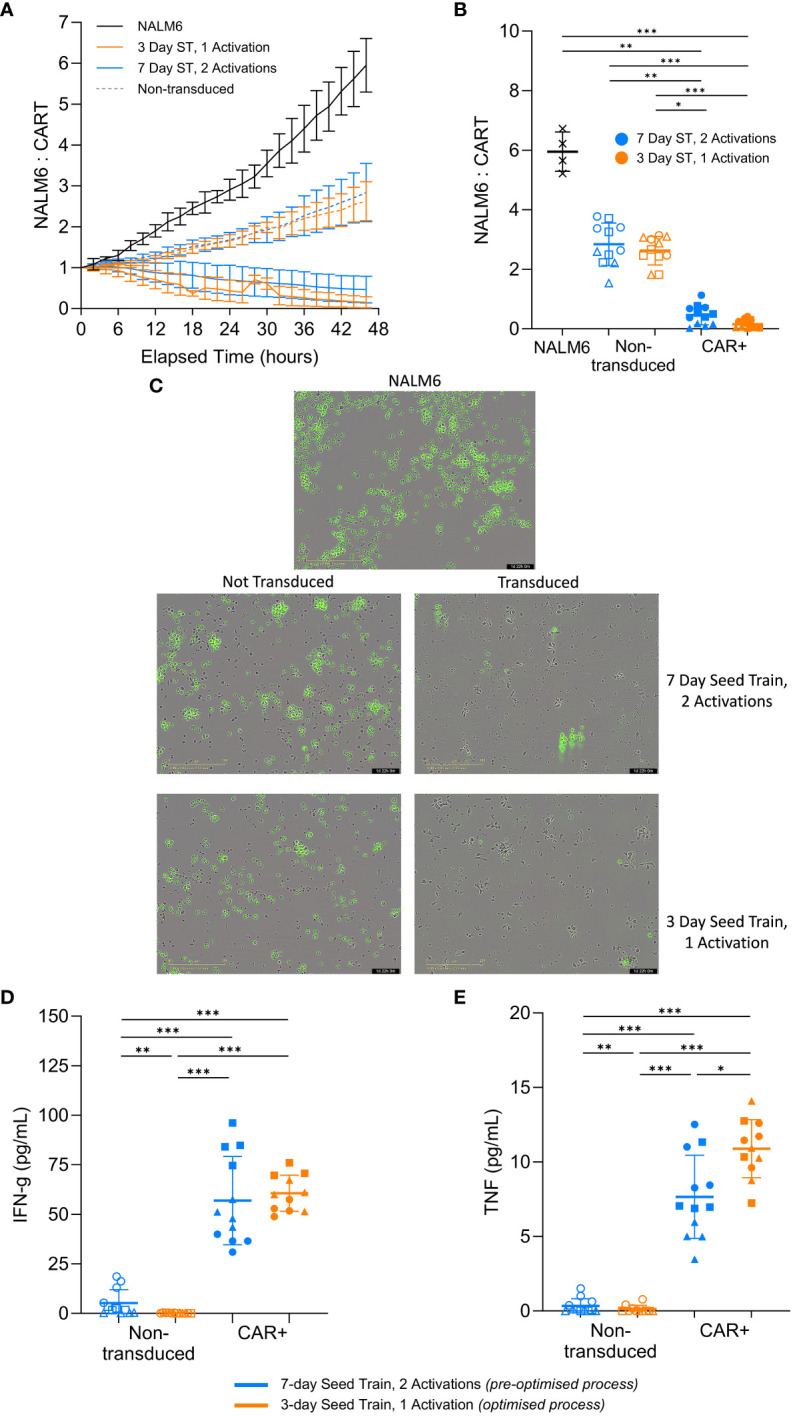
CAR-T cells effectively kill target cells and secrete cytokines post-expansion. Following expansion in flasks, cells were co-cultured with target GFP-positive NALM6 cells. Relative number of NALM6 cells during the 2-day co-culture **(A)**. Final relative number of NALM6 cells **(B)** and representative images of each culture type (20X magnification) **(C)** at the end of the 2-day co-culture. Cytokine levels in the medium for IFN-γ **(D)** and TNF-α **(E)** at the end of the 2-day killing assay co-culture. Each condition completed in quadruplicate (n=4). Points represent each donor (square = HD1, triangle = HD2, circle = HD3). Error bars represent standard deviation. One-way ANOVA and Dunn’s test completed where * is p<0.05, ** is p<0.01, *** is p<0.001.

In the experimental conditions with CAR+ cells, the normalized target NALM6 cell count decreased below 1 over time, indicating the target cells were being killed. Whereas, the killing assay controls without effector cells (NALM6 target cells only) or with effector cells that were not transduced with the CAR had normalized target cell counts that increased well above 1, indicating continued growth of the target NALM6 cells (**Figure 6A, B**). This suggests the CAR+ cells from both the pre-optimised and optimised conditions are preventing growth of the target cells and effectively killing them, with improved cytotoxic capacity demonstrated in the cells generated using the optimised process. The killing of the green, GFP-positive, target cells can be observed in representative images for each condition type after 2 days of co-culture. Images of the transduced conditions show very few target cells remaining. Additionally, the unlabelled CAR+ cells have expanded and elongated which indicates the T cells are activated and in a killing state (**Figure 6C**). The results from the cytotoxicity assay suggests CAR-T cells generated using the optimised process retain the functional capacity to kill target cells *in vitro.*

Functional T cells produce cytokines, such as IFN-γ and TNF-α, to initiate and amplify the overall immune response to a target cell (34). Both of the CAR+ conditions produced significantly more (p>0.05) IFN-γ and TNF-α compared to the non-transduced controls at the end of the 2-day co-culture (**Figure 6D, E**). Interestingly, the pre-optimised process condition produced similar levels of IFN-γ (p>0.05) but significantly less TNF-α (p=0.04) compared to the more optimised condition (**Figure 6D, E**). While this is not a large difference, the general trend is notable.

### 3.4 Validating the optimised conditions for scalable CAR-T cell expansion in automated stirred-tank bioreactors

**Figure 7:**
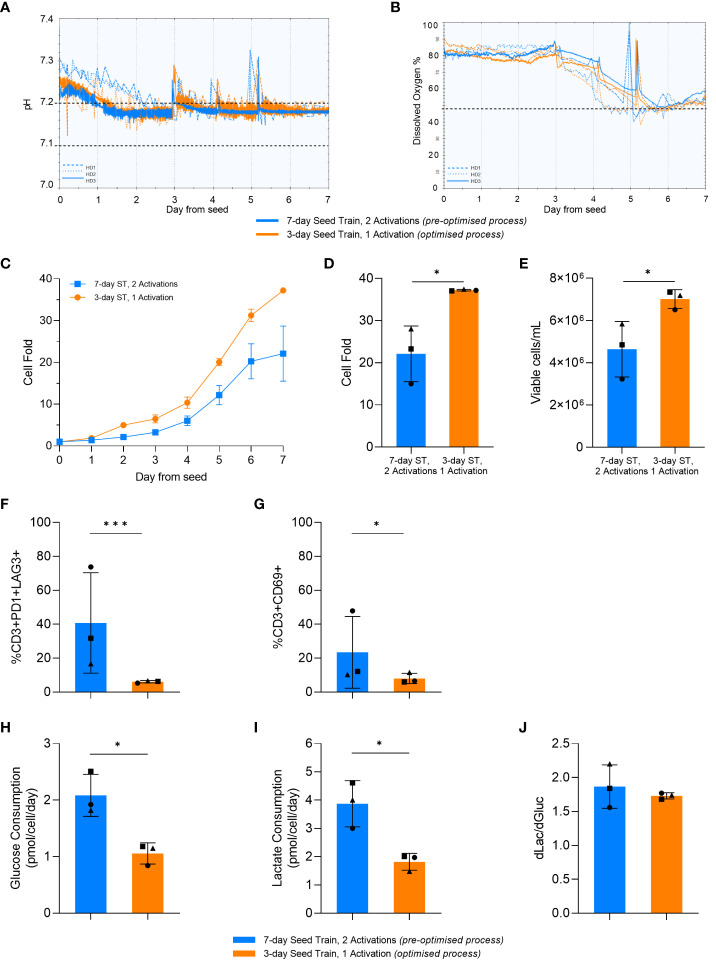
Optimised process increased cell expansion in the stirred-tank bioreactor (STR). pH **(A)** and dissolved oxygen (DO) **(B)** over time in the STR cultures. Dashed lines represent HD1, dotted lines represent HD2, and solid lines represent HD3. Horizontal black dashed lines represent the parameter setpoints. Cell fold by day in the STR **(C)**. Cell fold **(D)**, cell density **(E)**, T-cell exhaustion **(F)** and activation **(G)** on day 7 of STR culture. Average glucose consumption **(H)**, lactate production **(I)**, and change in lactate divided by change in glucose (dLac/dGluc) **(J)**. Day 0 represents the day STRs were seeded post seed train (ST). Points represent each donor (square = HD1, triangle = HD2, circle = HD3). Error bars represent standard deviation. Statistical pooled t-test analyses completed where * is p<0.05, *** is p<0.001.

The final phase of work aimed to compare the optimised and pre-optimised processes by investigating the impact of the upstream expansion process parameters when translated into the Ambr^®^ 250 High Throughput stirred tank bioreactor (STR) process. To support a more consistent STR culture environment, pH and DO were successfully controlled within the desired setpoint ranges (**Figure 7A, B**).

In alignment with the results from the previous DOE and small-scale studies, the optimised upstream process significantly increased cell fold in the bioreactor (p=0.017) (**Figure 7C, D**). This resulted in a significant increase in the final cell density at the end of the bioreactor culture using the optimised process in comparison to the pre-optimised process, with 4.6e6 ± 1.3x10^6^ cells/mL and 7.1e6 ± 0.4x10^6^ cells/mL generated in the pre-optimised and optimised process conditions respectively (**Figure 7E**).

Similar to the previous DOE and small-scale studies, there was no significant difference (p>0.05) in differentiation between the pre-optimised and optimised process in the STR. Moreover, as observed with the previous studies, there was a lower expression of exhaustion markers observed in the STR after the optimised upstream process, with only 8 ± 3% of cells being CD3+PD1+LAG3+. The percentage of CD3+PD1+LAG3+ cells in the bioreactor culture that used the pre-optimised upstream process was more variable compared to the optimised process (p=0.03) with standard deviations of 29.6% and 0.7%, respectively. For each individual donor, the optimised upstream process resulted in a lower percentage of CD3+PD1+LAG3+ cells compared to the pre-optimised upstream process in the STR. This trend aligns with the previous results (**Figure 7F**), with a similar trend being observed with the expression of CD69 at the end of culture (**Figure 7G**).

Furthermore, the STR cultures with the pre-optimised upstream process consumed nearly double (p=0.01) the amount of glucose per cell compared to cultures with the optimised upstream process (**Figure 7H**). The optimised upstream process also produced nearly half the amount of lactate per cell compared to the pre-optimised cultures (p=0.015) (**Figure 7I**). Both STR cultures had a similar (p>0.05) ratio of glucose consumed to lactate produced with about 2 molecules of lactate being produced for every glucose molecule consumed (**Figure 7J**). This is indicative of the majority of the cells using aerobic glycolysis for energy production, in alignment with the results observed in flasks.

## 4 Discussion

### 4.1 Cell yield is impacted by critical process parameters

This study highlights the impact of critical process parameters on CAR-T cell yield and critical quality attributes. Cell yield is an important response to monitor as it is essential to maximise the number of CAR-T cells produced. In the context of an autologous CAR-T therapy, a higher cell yield allows the manufacturing time to be decreased. For an allogeneic CAR-T therapy, a higher cell yield increases the number of doses produced per batch which can help decrease manufacturing costs. The results of this work demonstrated that repeated activations, a longer seed train, and a higher seeding cell density can all negatively impact cell yield.

The process involving two activation steps was shown to have the largest detrimental impact on CAR-T growth and CQAs compared to the other experimental factors tested. In a study completed by 23, the negative effect on growth after re-activation in combination with a long culture prior to re-activation was also shown. However, the effect of each individual factor was unclear in this study (23). This DOE study was able to show the negative effect of repeated activations on growth is stronger than longer culture time. Whilst it is unlikely that repeated activations will be required for autologous CAR-T production, the extended culture time to generate higher cell yields in the context of allogeneic CAR-T manufacture may result in companies adopting a strategy of activating more than once. However, our findings clearly demonstrate the detrimental impact that arises from multiple activations and fundamentally undermines the intended goal of generating higher cell yields. Therefore, for allogeneic applications, attempts to increase cell yield should focus on other process improvements instead.

Our findings also demonstrated that a longer seed train time and higher seeding density also negatively impact cell growth but to a lesser extent than repeated activations. The impact of a longer seed train was greater than the seeding density, particularly when the cells were activated twice. Conversely, 25 reported better cell yield with a longer culture time that was greater than 3 weeks compared to a shorter culture time, however, the growth rates over time were not directly compared, only the final cell yield (25). If the cells are starting to slow in terms of their growth rate, this would be sub-optimal for *in vivo* administration with evidence to suggest that this would lead to lower engraftment and CAR-T cell persistence (35).

### 4.2 Cell exhaustion impacted by critical process parameters

Exhausted cells will exhibit decreased growth and function (36). This is likely why exhausted cells have been associated with decreased clinical efficacy for CAR-T cell therapies (33). A T cell is usually classified as exhausted when there is sustained expression of multiple inhibitory markers, such as PD1 and LAG3 (37). A T cell is considered late stage exhausted when additional markers, such as CD69 which is normally an activation marker, start to also become constitutively expressed (36). Therefore, it is essential to prevent this truly exhausted state during CAR-T manufacturing. The DOE conditions that were repeatedly activated and had a long seed train showed high expression of PD1, LAG3, and CD69 (**Figure 3**). Therefore, these T cell populations were likely exhausted.

The lower secretion of TNF-α in the *in vitro* killing assay by the pre-optimised process that had the longer, 7-day seed train and was activated twice also indicates the cells were likely more exhaustion compared with the optimised process. Exhausted T cells will typically start to produce less cytokines. The cells will first exhibit decreased production of TNF-α. As the cells become more dysfunctional, they will start to produce less IFN-γ and will ultimately show lower clinical efficacy (36, 38).

The observed differences in metabolic kinetics in this work are likely connected to the functional state of the cells. Typically, higher glucose consumption is associated with increased cell growth (15). This is because T cell activation as well as exposure to IL-2 will push the cells towards effector functions. The activated cells will then utilise glycolysis and other downstream metabolic pathways to support cell proliferation (39, 40). However, even though the conditions which included two activations had a higher glucose consumption rate, they did not have a higher growth rate. The conditions did produce close to the theoretical 2 mol lactate per 1 mol of glucose that is associated with glycolysis (31, 32). This indicates the activated cells are actively using glycolysis. However, there are likely inefficiencies in the downstream pathways, such as for nucleotide production, that are essential for the proliferation of cells. Metabolic inefficiencies like these are associated with exhausted T cells (34, 38). The conditions that were activated twice had high exhaustion marker expression (**Figure 3**), therefore these observed metabolic inefficiencies are likely associated with the cells being exhausted.

### 4.3 Critical process parameter robustness to donor and expansion platform

The comparisons of the optimised and pre-optimised processes with multiple donors and in the stirred tank bioreactor highlight that the impact of these process parameters on cell yield are independent of donor and expansion platform. It is also important to note that even though the optimised conditions achieved higher cell densities, the cells remained minimally differentiated. This indicates the potentially higher levels of cell signalling in the medium due to denser cells did not push the cells to differentiate. Cells in the naïve and central memory differentiation states have been associated with greater clinical efficacy for CAR-T cell therapies (33, 41). The high percentage of less differentiated central memory populations in these experiments is therefore desirable.

Low exhaustion marker expression with the optimised process with a shorter seed train and only one activation was also shown to be independent of donor and expansion platform. This is particularly notable since the bioreactor culture concomitantly achieves higher cell densities. Exhaustion marker expression in normal circumstances serves as a negative feedback loop for cell growth to prevent over activation and growth (36, 42). Therefore, it would be expected that the higher cell densities in the bioreactor would increase the expression of these exhaustion markers. This lower exhaustion marker expression in the bioreactor could be due to the agitated culture, which would maintain the effective cell density lower compared to the flask culture. This suspension could, therefore, be diluting the cell signalling that could push the cells towards an exhausted state.

These results also highlight how the impact of donor variation can be dependent on process parameters and expansion platform. Donor variation is known to be a significant challenge for CAR-T cell therapies (29, 43). However, these findings show that despite the inherent donor variability that will be experienced, in particular for autologous production, process optimisation can still be achieved with the findings translating across multiple donors.

## 5 Conclusions

This study demonstrates a systematic approach and methodology using DOE for CAR-T cell culture CPP optimisation. It is critical to optimise and understand the impacts of each CPP as these can significantly alter CAR-T cell product CQAs. The initial DOE experiment highlighted process parameters can have combined effects on cell characteristics. Shorter seed train times and a single resulted in improved growth, viability, exhaustion marker expression and metabolism. It was notable that repeated activations dominated the effect on cell responses. Conditions identified in the DOE that resulted in the highest cell yield and quality versus the lowest cell yield and quality were subsequently investigated with multiple donors in different expansion platforms. The optimised range of CPPs improved cell growth and exhaustion marker expression whilst maintaining killing function in all donors tested, and when scaled-up to the stirred tank bioreactor expansion system.

Overall, this study showed it is critical to understand the manufacturing design space and select a robust process for the production of CAR-T cell therapies. These results indicate understanding and controlling more of the process can improve CQAs that are linked to clinical efficacy. This work also showed the feasibility of initially optimising upstream process parameters at small scale to reduce costs prior to scaling up into stirred-tank bioreactors. Implementing more control into the manufacturing process could, therefore, help improve the reproducibility of these products.

## Data availability statement

The original contributions presented in the study are included in the article/**Supplementary Material**. Further inquiries can be directed to the corresponding author.

## Ethics statement

Ethical approval was not required for the studies on humans in accordance with the local legislation and institutional requirements because only commercially available established cell lines were used.

## Author contributions

TH: Conceptualization, Data curation, Formal analysis, Investigation, Methodology, Software, Visualization, Writing – original draft, Writing – review & editing. FS: Conceptualization, Formal analysis, Methodology, Project administration, Resources, Supervision, Validation, Writing – review & editing. VS: Conceptualization, Formal analysis, Methodology, Software, Writing – review & editing. WG: Conceptualization, Formal analysis, Methodology, Software, Supervision, Validation, Writing – review & editing. TS: Formal analysis, Methodology, Software, Supervision, Validation, Visualization, Writing – review & editing. NB: Conceptualization, Formal analysis, Methodology, Software, Supervision, Visualization, Writing – review & editing. QV: Conceptualization, Formal analysis, Investigation, Project administration, Resources, Supervision, Writing – review & editing. JH: Formal analysis, Methodology, Project administration, Writing – review & editing. PS: Data curation, Formal analysis, Investigation, Methodology, Visualization, Writing – review & editing. ND: Formal analysis, Investigation, Methodology, Project administration, Resources, Supervision, Writing – review & editing. QR: Conceptualization, Formal analysis, Funding acquisition, Investigation, Methodology, Project administration, Resources, Supervision, Validation, Visualization, Writing – original draft, Writing – review & editing.

## Supplementary material

Supplementary Figure 2 Model for population doublings (PD) on day 7 of G-Rex^®^ culture. Overview of model terms and p-values **(a)**. Observed vs predicted values for PD on day 7 in the G-Rex^®^ **(b)**.

Supplementary Figure 3 Model for CD3+PD1+LAG3+ exhaustion marker expression of T-cells on day 7 of G-Rex^®^ culture. Overview of model terms and p-values (a). Observed vs predicted values for exhaustion marker expression on day 7 in the G-Rex^®^.

Supplementary Figure 4 Model for CD3+CD69+ activation marker expression of T-cells on day 7 of G-Rex^®^ culture. Overview of model terms and p-values **(a)**. Observed vs predicted values for act marker expression on day 7 in the G-Rex^®^.
