# Supplementary figures and images for: A quality-by-design approach to improve process understanding and optimise the production and quality of CAR-T cells in automated stirred-tank bioreactors
Source: Front Immunol. 2024 Apr 9;15:1335932. doi: 10.3389/fimmu.2024.1335932 (PMC11035805; doi:10.3389/fimmu.2024.1335932)

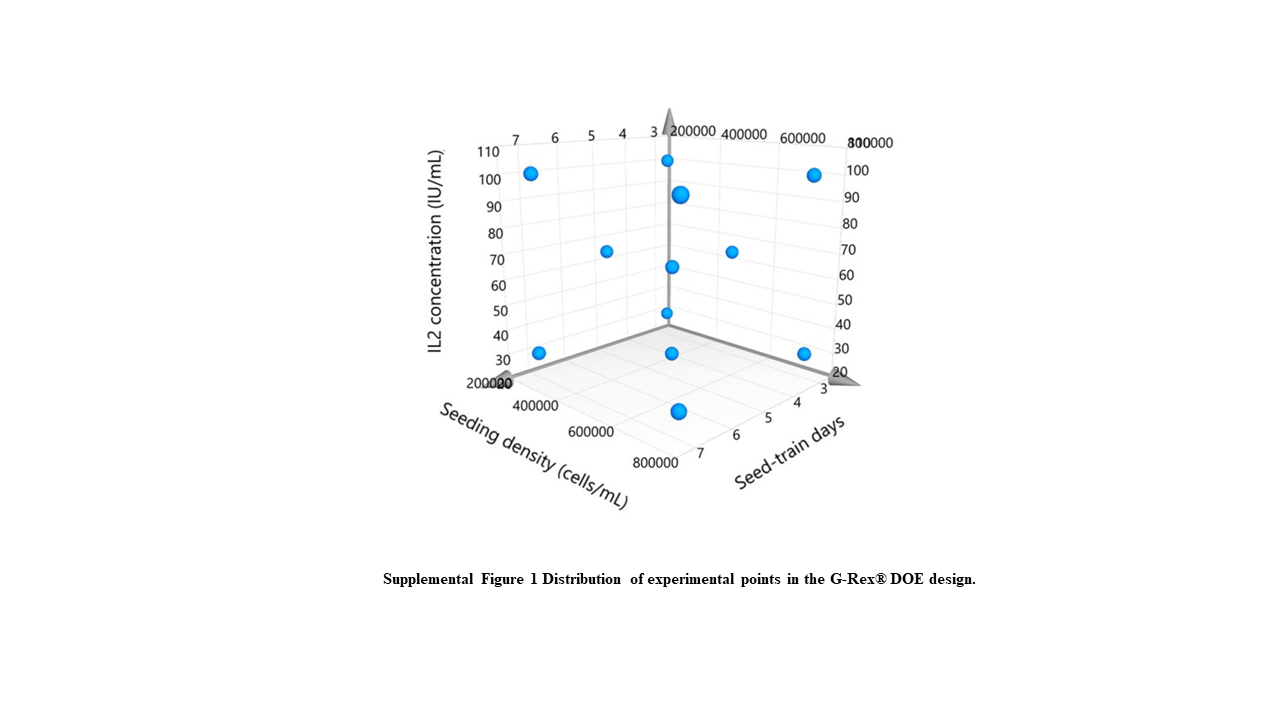

Supplement: Supplementary file 1 [file Image_1.tif]

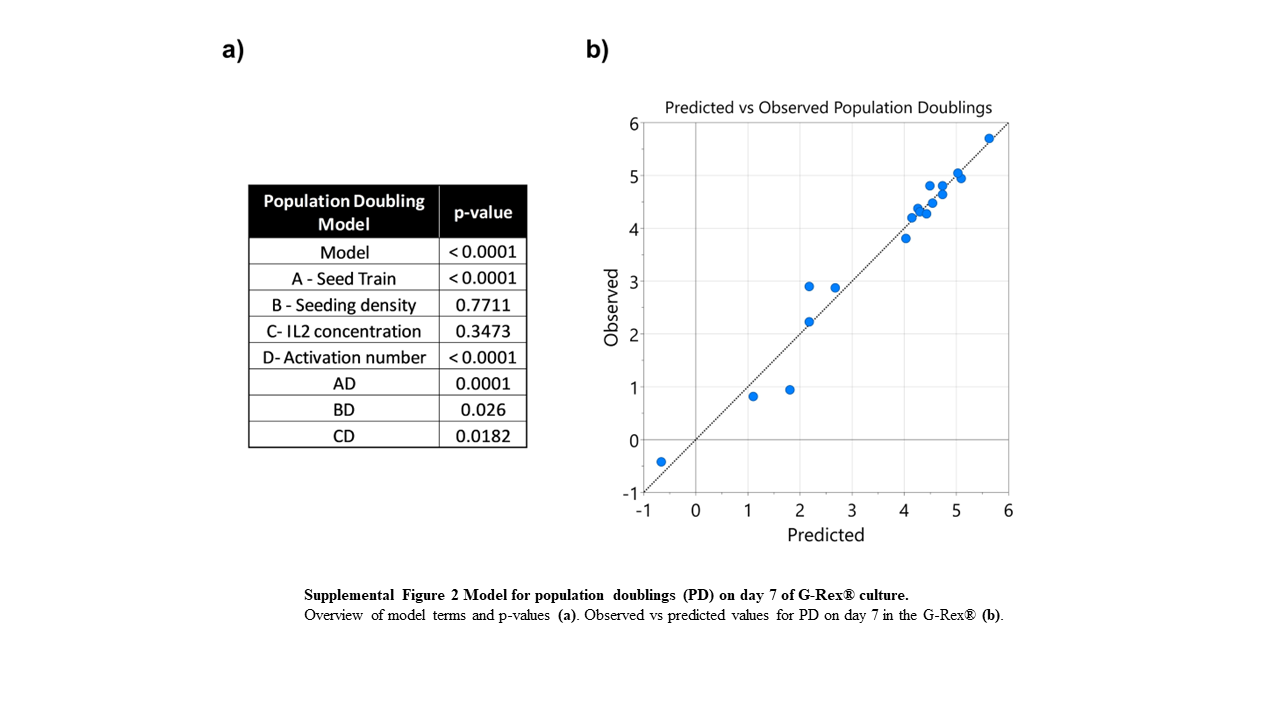

Supplement: Supplementary Figure 2 — Model for population doublings (PD) on day 7 of G-Rex® culture. Overview of model terms and p-values (a). Observed vs predicted values for PD on day 7 in the G-Rex® (b). [file Image_2.tif]

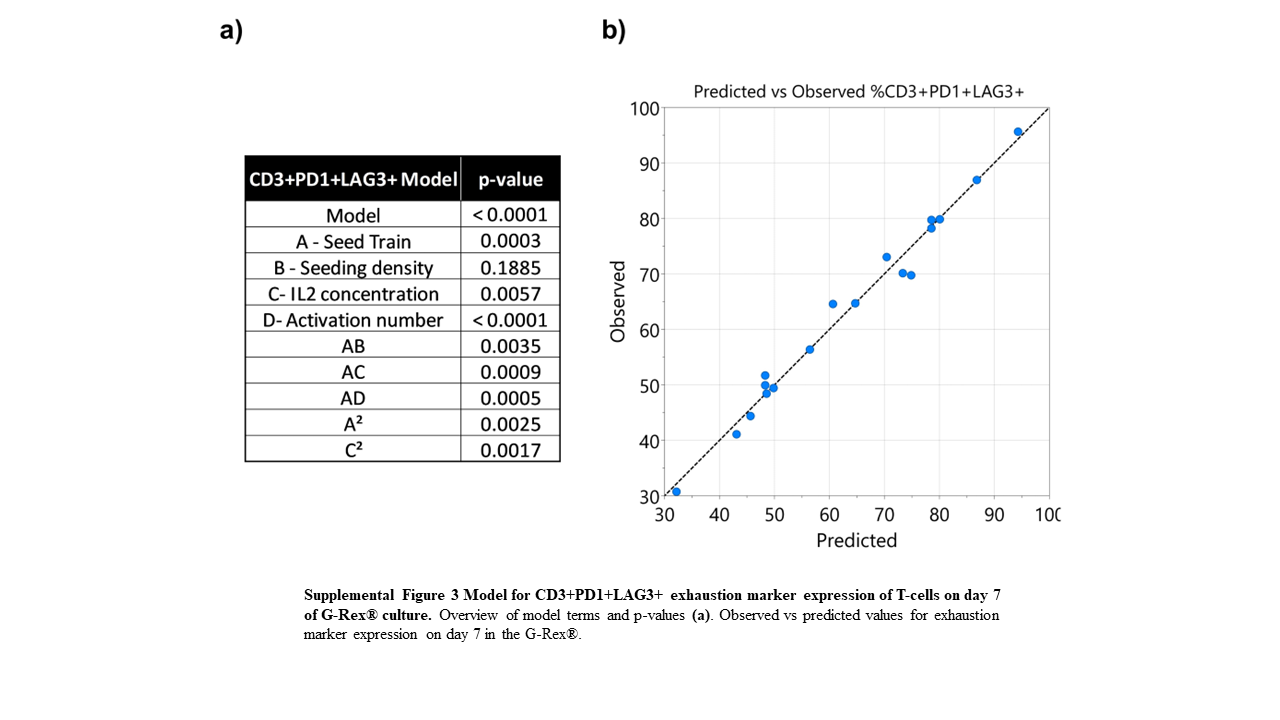

Supplement: Supplementary Figure 3 — Model for CD3+PD1+LAG3+ exhaustion marker expression of T-cells on day 7 of G-Rex® culture. Overview of model terms and p-values (a). Observed vs predicted values for exhaustion marker expression on day 7 in the G-Rex®. [file Image_3.tif]

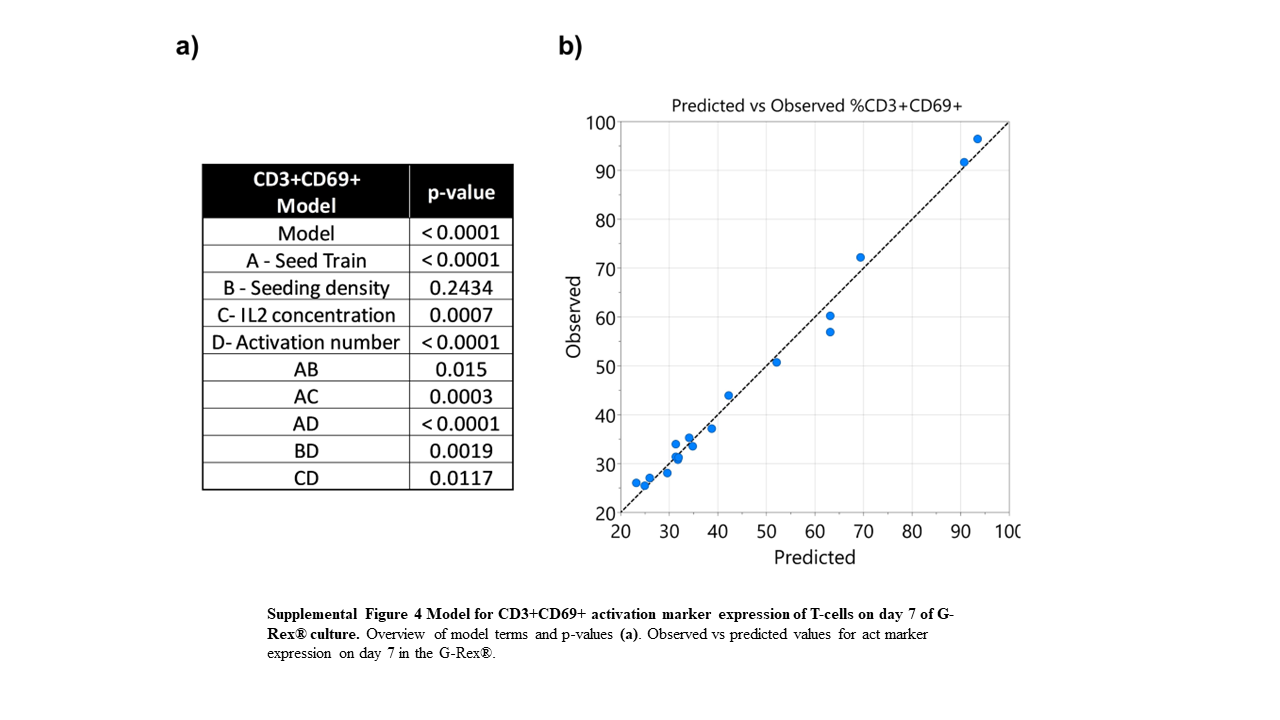

Supplement: Supplementary Figure 4 — Model for CD3+CD69+ activation marker expression of T-cells on day 7 of G-Rex® culture. Overview of model terms and p-values (a). Observed vs predicted values for act marker expression on day 7 in the G-Rex®. [file Image_4.tif]
